# Electrical Stability and Piezoresistive Sensing Performance of High Strain-Range Ultra-Stretchable CNT-Embedded Sensors

**DOI:** 10.3390/polym14071366

**Published:** 2022-03-28

**Authors:** Hammad R. Khalid, Daeik Jang, Nadir Abbas, M. Salman Haider, Syed N. A. Bukhari, Cyrus R. Mirza, Noureddine Elboughdiri, Furqan Ahmad

**Affiliations:** 1 Civil and Environmental Engineering Department, King Fahd University of Petroleum & Minerals, Dhahran 31261, Saudi Arabia; 2 Interdisciplinary Research Center for Construction and Building Materials, King Fahd University of Petroleum & Minerals, Dhahran 31261, Saudi Arabia; 3 Department of Civil and Environmental Engineering, KAIST, 291 Daehak-ro, Yuseong-gu, Daejoen 34141, Korea; svs2002@kaist.ac.kr; 4 Chemical Engineering Department, College of Engineering, University of Ha’il, P.O. Box 2440, Ha’il 81441, Saudi Arabia; n.abbas@uoh.edu.sa (N.A.); n.elboughdiri@uoh.edu.sa (N.E.); 5 Department of Chemical Engineering, University of Gujrat, HH Campus, Gujrat 50700, Pakistan; salman.haider@uog.edu.pk; 6 Department of Pharmaceutical Chemistry, College of Pharmacy, Jouf University, Sakaka, Al-Jouf 72341, Saudi Arabia; sbukhari@ju.edu.sa; 7 Department of Civil Engineering, College of Engineering, University of Ha’il, Ha’il 81441, Saudi Arabia; cy.mirza@uoh.edu.sa; 8 Chemical Engineering Process Department, National School of Engineers Gabes, University of Gabes, Gabes 6029, Tunisia; 9 Department of Mechanical and Mechatronics Engineering, Dhofar University, Salalah 211, Oman; fahmad@du.edu.om

**Keywords:** stretchable senor, flexible sensor, wearable sensor, functional composite, carbon nanotubes, piezoresistive material, polymeric composite, three-roll mill machine

## Abstract

Highly flexible and stretchable sensors are becoming increasingly widespread due to their versatile applicability in human/robot monitoring sensors. Conductive polymeric composites have been regarded as potential candidates for such sensors, and carbon nanotubes (CNTs) are widely used to fabricate such composites. In the present study, CNT-embedded high flexible sensors were fabricated using a facile three-roll milling method, which mitigates the drawbacks of the conventional fabrication methods. CNTs content varied between 0.5 and 4.0 wt.%, and the percolation threshold range was obtained via conductivity/resistivity values of the fabricated sensors. Following this, the electrical stability of the sensors was examined against the various DC and AC signals. Furthermore, the fabricated sensors were stretched up to 500% strain, and their sensitivity against varying strain amplitudes was investigated in terms of the change in resistance and gauge factors. Lastly, the fabricated sensors were applied to human fingers for monitoring finger bending and releasing motions to validate their potential applications. The experimental results indicated that these sensors have a percolation threshold of around 2% CNTs content, and the sensors fabricated with 2 to 4% CNTs content showed measurable resistance changes against the applied strain amplitudes of 50–500%. Among these sensors, the sensor with 2% CNTs content showed the highest sensitivity in the studied strain range, exhibiting a resistance change and gauge factor of about 90% and 1.79 against 50% strain amplitude and about 18,500% and 37.07 against 500% strain amplitude, respectively. All these sensors also showed high sensitivity for finger motion detection, showing a resistance change of between 22 and 69%.

## 1. Introduction

Flexible and stretchable sensors have been attracting attention to meet the requirements for versatile applications such as wearable electronic devices, bio-medical sensors, and monitoring motions of humans and robots [1,2,3]. Such sensors are required to possess high flexibility and sensitivity to broaden their applicability [4]. For these reasons, conductive polymeric composites are regarded as potential candidates to be utilized as such sensors [5]. Many types of conductive fillers have been introduced to fabricate the conductive polymeric composites; however, the carbon nanotubes (CNTs) are found to be favorable conductive fillers due to their outstanding mechanical and piezoresistive sensing properties [6,7,8].

Many attempts have been made to fabricate the CNT-embedded flexible sensors. For example, Jang et al. [9] added CNTs into magneto-rheological elastomers for ensuring proper electrical conductivity to be used as flexible sensors. Cui et al. [10] fabricated flexible pressure sensors using a 3D CNT-embedded sponge, and it showed high sensitivity for human-to-computer interactions. Nevertheless, the dispersion of CNT particles homogeneously into the polymeric matrix is still a difficult task. Various dispersion methods, including spray-deposition [11], hot press [12,13], inkjet printing [14], three-roll milling [15], and ultra-sonication with dispersant [16], have been reported in the literature. Lipomi et al. [17] sprayed a CNT solution on PDMS layers, serving as a conductive layer. The prepared sensors were stretched up to 150% strain and showed good conductivity. Jang et al. [18] used ultra-sonication with polymeric dispersant and the mechanical mixing method for improving the dispersion of the CNTs. The sensors showed good sensing behavior in the studied strain range, i.e., up to 100% strain. Zhang et al. [19] fabricated monofilament CNTs/silicone polymer sensors. Sensors showed a sensing response up to 1300% strain and good stability under 100% strain. Amjadi et al. [20] fabricated ultra-stretchable CNTs/silicone polymer composites. The sensors were stretched up to 500% strain and showed a good sensing response. However, some drawbacks were noticed in the fabrication methods used in these studies, such as the use of high amounts of solvents, health risks associated with their evaporation into the environment, complexity, and high costs, which makes them non-favorable methods for mass production [21]. Alternatively, fabrication using a three-roll milling machine is simple, fast, compatible with industrial techniques, and requires minimal use of solvents [15]. Hence, the three-roll milling machine method was used in this study to fabricate the wide strain range (up to 500%) ultra-stretchable CNT-embedded composite sensors.

Although the fabrication of CNT-embedded polymeric composites using a three-roll milling machine has been reported in the literature [22], few efforts have been made to investigate the electrical stability under DC and AC signals with various levels of voltages and frequencies of such sensors. In addition, comprehensive studies investigating the electrical stability, piezoresistive sensing performances, and potential applicability in monitoring human motion are rarely reported, to the best of the authors’ knowledge. However, it is important to observe the electrical stability under various signals (e.g., DC and AC signals) to enhance the possibility of using such CNT-embedded polymeric sensors in real industrial applications, e.g., human and/or robot motions monitoring. Therefore, this paper aims to use the facile fabrication method using a three-roll milling machine and investigate the electrical stability and piezoresistive sensing performances of the fabricated sensors comprehensively. Based on these purposes, this paper is organized as follows. First, the three-roll milling machine method was used to fabricate the CNT-embedded flexible composite sensors. Since the sensitivity of the sensors is vitally affected by the percolation threshold of the CNT-embedded sensors [23], sensors incorporating various CNTs amounts (0.5–4 wt.%) were fabricated. Then, electrical characteristics including conductivity and resistivity values of the fabricated sensors were measured to determine the percolation threshold range. Additionally, the electrical stability of the selected sensors, based on the percolation threshold, was systematically studied under the application of DC and AC signals, respectively. Following this, the sensors were subjected to cyclic strain loadings to investigate their piezoresistive sensing responses against increasing strain amplitudes, i.e., 0–500%. Based on the piezoresistive sensing test results, the sensitivity of each sensor expressed as a gauge factor was investigated. Lastly, the sensors were attached to a human finger to validate their potential use in human-movement sensing.

## 2. Materials and Methods

### 2.1. Materials and Specimens Details

The two parts (*A* and *B*) of silicone polymer (Ecoflex^®^ 00-30) were procured from Smooth-on Inc. (Macungie, PA, USA). Parts A and B of the polymer were used at a 1:1 mass ratio as recommended by the manufacturer. The polymer had a tensile strength of 200 psi, a specific gravity of 1.07, and an elongation-at-break value of 900%. The multi-walled CNTs, having a specific gravity, diameter, and length of 1.32, 10–40 nm, and 10 μm, respectively, were received from Hyosung Inc. (Seoul, Korea). The CNTs showed the presence of a defect or carbonaceous compounds in Raman spectra [24]. The acetone was procured from Carlo Erba Reagents. The CNTs content varied from 0 to 4% (0.5, 1, 1.5, 2, 3, and 4%) to investigate the effects of CNTs content on the stretchability and sensitivity of the sensors against various strain levels. The details of the sensors are given in Table 1.

### 2.2. Fabrication Details

**Figure 1:**
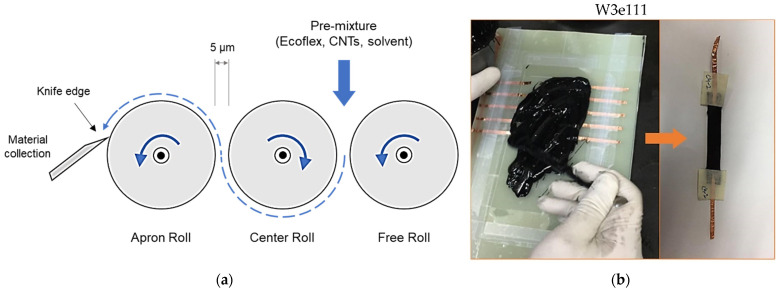
Preparation of CNT-embedded sensors: (**a**) Schematic of mixture passing from three-roll milling machine; (**b**) prepared specimen.

A three-roll milling machine was used for uniform dispersion of CNTs in the polymer matrix as shown in Figure 1. After initial hand mixing of the measured amounts of the polymer and CNTs, the mixture was passed through rollers, rotating at 200 rpm speed with a gap of 5 μm. A small amount of acetone (2–3% of total mixture mass) was added to reduce the initial viscosity of the mixture. Each mixture was passed through the rollers five times, following the recommendations of the literature [15]. The final mixture was poured in pre-prepared molds and kept for curing at 70 °C for 2 h. Lastly, 10 × 75 mm^2^ individual rectangular specimens were obtained via cutting with a paper cutter (Figure 1b).

Molds were prepared using a scotch-tape-lined composite plate substrate and 1-mm-thick glass slides. The glass slides were taped at the edges of the substrate to obtain a rectangular pouring area of approximately 120 × 75 mm^2^ (Figure 1b). The 3-mm-wide copper tape strips, which served as electrodes, were also attached on both sides of the pouring area (along the length) at 10 mm spacing, hence the cured composites had embedded electrodes on both ends, as shown in Figure 1b.

### 2.3. Electrical Characterization

**Figure 2:**
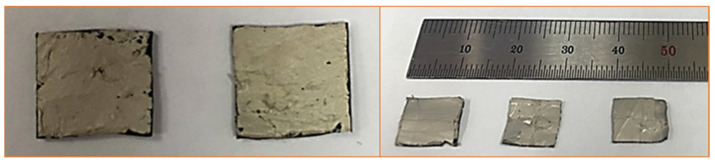
Prepared samples for resistivity measurement.

The electrical conductivity (σ) and resistivity (ρ) of the sensors were measured following the method reported in the literature [25]. First, 10 × 10 mm^2^ square specimens (1 mm thick) were cut from the prepared composites. Both sides of the specimens were fully covered with silver paste to serve as electrodes, as shown in Figure 2. One electrical probe was then attached to each side of the specimen, and resistance values were recorded using a portable multimeter. Conductivity and resistivity were then calculated using Equation (1) [13,25]. For each composite type, three such specimens were used, and average conductivity and resistivity values are reported.

(1) σ=1ρ=LRA

where *R* is the measured average resistance of the samples, *A* is the area of the electrode (=100 mm^2^), and *L* is the distance between the electrodes (=1 mm).

To investigate the electrical stability of the fabricated sensors, DC input voltage in the range of 3 to 15 V was applied to the composites using a power supply (PL-3005 S), and the electrical DC current flowing through the composites was measured. Then, the relationship between the electrical DC current and input voltage was examined. Furthermore, an LCR meter (Hioki, Nagano, Japan, IM3523) was used to measure the electrical impedance under the AC signals with different input frequencies (50 to 500 Hz).

### 2.4. Cyclic Stretching Test

The electrical characteristics of the prepared composites were analyzed by means of tensile cyclic loadings under a micro material testing machine (Instron 5848). The specimens were subjected to incremental cyclic loadings of up to 500% strain, i.e., 50%, 100%, 200%, 300%, 400%, and 500%. Starting from 0 to 50% strain, three cycles were applied at each strain level before increasing it to the next strain level. The loading rate was kept constant at 0.5 mm/s.

### 2.5. Human-Motion Sensing

The prepared composites were also used for detecting movements of human joints for their possible use in wearable electronic devices. The rectangular specimens were attached to the index finger, and their sensing response against bending movements was recorded.

## 3. Results and Discussion

### 3.1. Electrical Characteristics and Stability

**Figure 3:**
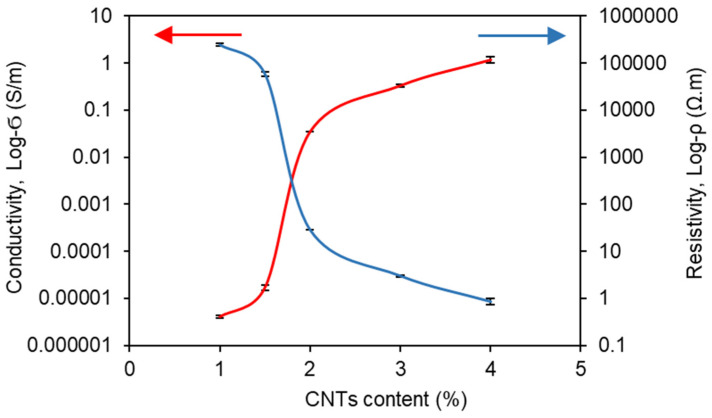
Electrical conductivity and resistivity of the sensors fabricated with different CNTs content.

The conductivity and resistivity results are plotted in Figure 3. The C0.5 specimen with 0.5% CNTs did not exhibit any conductivity during the multimeter measurements. This was possibly due to the non-connectivity of CNTs, resulting from the low CNTs content in this specimen. Hence, this specimen was not considered for further analysis. The remaining specimens showed a clear increasing trend in conductivity with the increase in CNTs content from 1% to 4%. Specifically, a dramatic increase can be seen in the range of 1–2% CNTs content, which indicates the percolation threshold of these composites [18]. In the literature, a percolation threshold between 0.05 and 10 wt.% has been reported for various nanotubes-based thin-film composites [26], thus a percolation threshold of 2% is towards the lower end of the reported values. Moreover, Souri et al. [27] reported a percolation threshold of about 5% in CNTs/Polyurethane (PU) composites fabricated using a similar three-roll milling machine method. A lower percolation threshold in this study indicates that the dispersion of CNTs was possibly better in the silicone matrix compared to the PU matrix. Furthermore, it also shows that the three-roll milling method is appropriate for dispersing CNTs in the polymeric composites, avoiding the excessive use of solvents.

**Figure 4:**
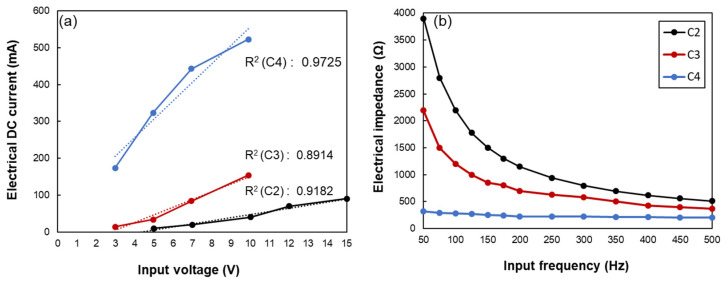
Relationship between (**a**) electrical current and input voltage, and (**b**) electrical impedance and input frequency obtained in C2, C3, and C4 sensors.

According to the results in Figure 3, the specimens incorporating higher than 1.5% of CNT (i.e., C2, C3, and C4 specimens) showed relatively high conductivity, which has potential for use as sensors. Thus, the C2, C3, and C4 sensors were chosen, and their electrical stability was investigated in this section. According to the previous studies, the CNT-embedded composites show a decrease in electrical resistivity with increasing input voltages, since the electrons can skip over even the individual CNTs particles that are not directly in contact with each other [28,29]. This phenomenon is known as the tunneling effect; thus, it is important to observe the electrical stability under the different input voltages. For these reasons, the electrical DC current flowing through each sensor under the different input voltages is investigated in Figure 4a. The linear relationship between the input voltage and electrical DC current can be seen in Figure 4a, showing R-squared values of 0.9182, 0.8914, and 0.9725 for C2, C3, and C4 sensors, respectively. Thus, it can be inferred that these sensors confirmed Ohm’s law, indicating excellent electrical stability [30]. In addition, it can be found that the electrical characteristics of the C2 sensor were stable in input voltages from 3 V to 15 V, and C3 and C4 sensors showed electrical stability under 3 V to 10 V of input voltage. Meanwhile, it should be noted that an input voltage larger than 10 V does not apply to the C3 and C4 sensors. It has been reported that when current flows through CNT-embedded composites, the electrical energy can be converted to heat energy, indicating Joule’s heating law [16,31]. The electrical stability is significantly affected by the temperature of the composites; thus, it is necessary to control the input voltage properly to prevent the composites from heating.

Meanwhile, the electrical impedance of the C2, C3, and C4 sensors is exhibited in Figure 4b. For C2 and C3 sensors, the electrical impedance was found to be frequency-dependent, i.e., the electrical impedance decreased as the input frequency of the signal increased. This result can be explained by the property of capacitance in the CNT-embedded composites [30,32]. It is the fact that the capacitance is inversely proportional to the input frequency, and thus, the results are in close agreement with the property of capacitance reported in the previous studies [30,32]. Interestingly, the extent of the decrease in electrical impedance decreased as the CNTs content increased and became negligible for the C4 sensor. This can be attributed to the establishment of a denser conductive network in the C4 sensor compared to that in the C2 and C3 sensors. It can be inferred that in the C4 sensor, conduction was primarily due to the direct contact between CNTs, and the tunneling effect was minimal. Similar findings have been reported in the literature that the CNT-composites showed frequency-dependent behavior at lower CNT dosages, which became independent of frequency at higher CNTs content [33]. Thus, the findings derived from this study show that as the conductive network becomes denser, the variation of electrical characteristics under the application of DC and AC signals decreases, which are in close agreement with previous studies [32,33].

### 3.2. Piezoresistive Sensing Responses of the Sensors against Cyclic Loading

**Figure 5:**
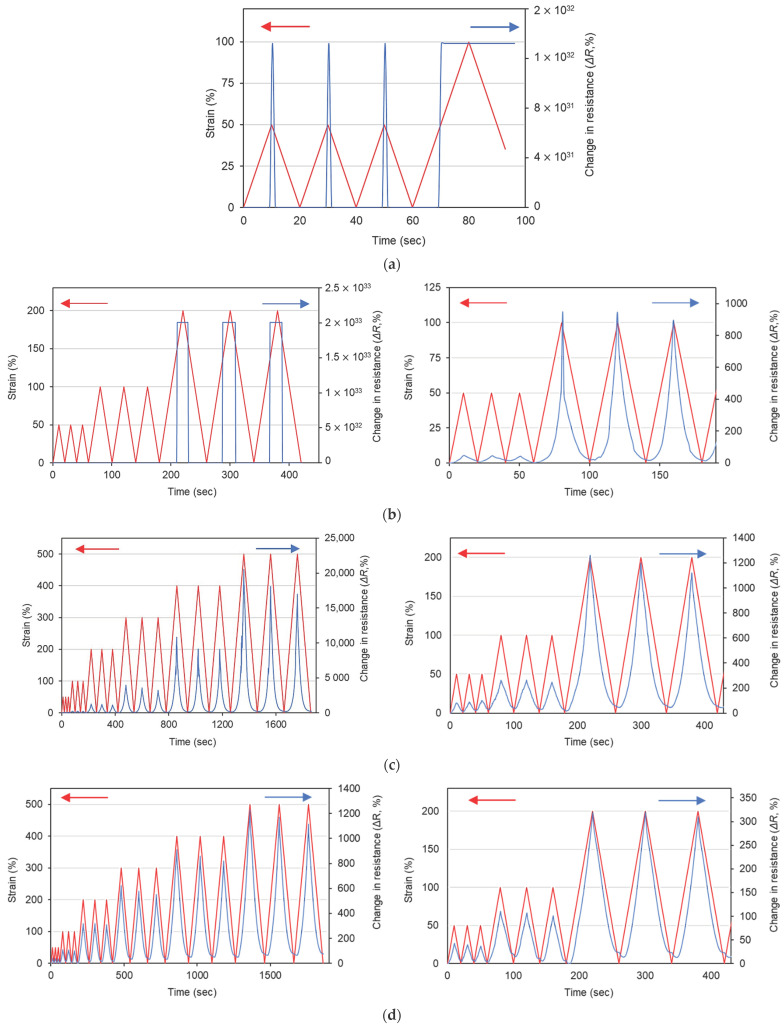

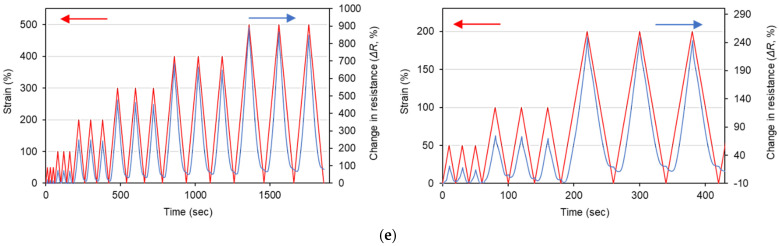
Cyclic loading vs. change in electrical resistance of sensors: (**a**) C1; (**b**) C1.5; (**c**) C2; (**d**) C3; (**e**) C4. Figures on right side are showing magnified images for lower strain levels.

The changes in the electrical resistance of the sensors against incremental cyclic loadings are plotted in Figure 5. The sensors with different CNTs contents showed distinct behavior at various strain levels. In general, the change in resistance of the sensors at a certain strain level decreased with the increase in CNTs content, i.e., the sensitivity decreased with an increase in CNTs content. Similar behavior is reported in the literature [34,35]. It is attributed to the fact that the inter-particle distance between CNTs is high near the percolation threshold, therefore conductivity is controlled by the tunneling effect [34,35]. As the CNTs content increases, direct contact between adjacent CNTs increases, which results in enhanced conductivity but relatively less sensitivity [34,35].

The resistance of the C1 sensor moved outside the measurable range of the multimeter at just 50% strain amplitude, as can be seen in Figure 5a. Since the 1% CNTs content was found to be below the percolation threshold of these composites considering conductivity data, the C1 sensor exhibited a complete loss of electrical paths at only 50% strain amplitude. In contrast, the C1.5 sensor exhibited measurable changes in resistance up to 100% strain amplitude. However, upon a further increase in the strain amplitude to 200%, the C1.5 sensor also showed the complete loss of the electrical path. These findings are in line with previous discussions about the tunneling effect and direct contact between CNTs. Since the CNTs content in specimens C1 and 1.5 was near the percolation threshold, they showed conductivity in an unstrained state. However, as these sensors were strained, the direct contact between CNTs, as well as the tunneling effect, decreased, resulting in a sharp increase in resistance and, ultimately, complete loss of electrical connections at 50% and 200% strains, respectively. In Figure 5, it should be noted that the initial electrical resistance increased as the number of loading cycles increased, regardless of the embedded CNT contents. This result is contributed to by the formation of conductive networks. As the loading cycle increases, the distances between the adjacent CNT particles increased compared to that prior to loadings [36]. In addition, the polymer matrix can also be stretched as the cyclic loading is applied. Therefore, the conductive networks can be disturbed, and it can increase the electrical resistivity of the sensors, thereby increasing the initial electrical resistances as the loading cycles increase.

**Figure 6:**
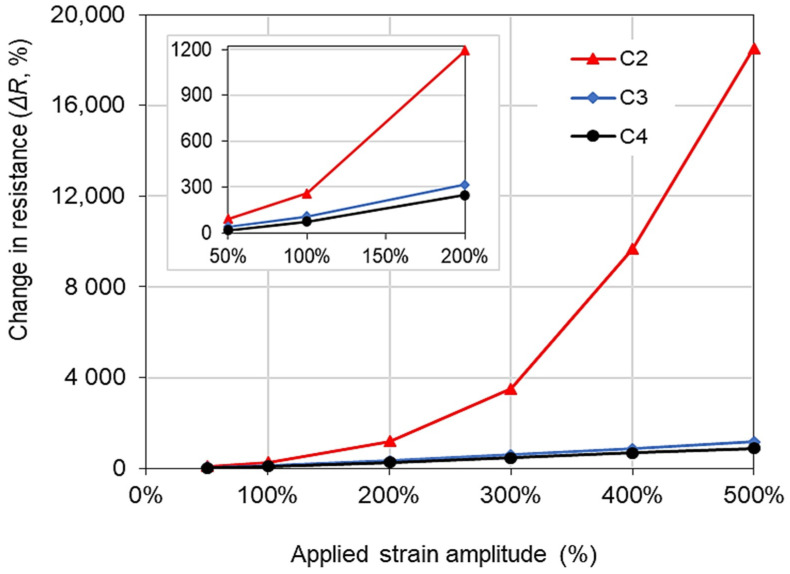
Relationship between the electrical resistance change and applied strain amplitudes.

All the remaining sensors with higher CNTs content (2–4%) showed measurable changes in electrical resistance up to 500% strain amplitude. This shows that these composites can be used for a wide range of high strains. For further analysis, the average changes in resistance at each strain level are plotted in Figure 6. The C2 sensor always exhibited a higher resistance change compared to the C3 and C4 sensors. For example, the C2 sensor showed an approximately 90% resistance change at the 50% strain level, which was higher than the 40% and 18% resistance changes obtained for the C3 and C4 sensors, respectively. As reported in a previous study [18], the strain variation caused by human motion is less than 100%. Thus, it can be said that the C2 sensor, showing high sensitivity in this strain range, can potentially be used for monitoring human motion. Furthermore, it can also be noted that at a higher strain level, it exhibited an exponential increase in the resistance change, showing an increased gauge factor at higher strain levels as has been reported in the literature [18]. Hence, it can be concluded that the 2% CNTs content is optimum among these specimens, showing high sensitivity for a wide range of strain amplitudes.

According to the cyclic loading test results in Figure 6, the sensitivity of the sensors expressed as the gauge factor is summarized in Table 2. Strain amplitudes equal to and less than 100% are regarded as the strain variation caused by human motion [18]. In this range, all the sensors showed excellent sensitivity, and in particular, the C2 sensor showed a gauge factor of 1.79 and 2.58 against the strain amplitudes of 50% and 100%, respectively. This gauge factor showed similar values compared to that obtained in the related literature [37,38,39]. Furthermore, C2, C3, and C4 sensors showed gauge factors of 37.04, 2.34, and 1.74 against 500% strain amplitude, respectively. As reported in previous literature, the gauge factors decreased as the loading cycles increased due to the deformation of the matrix and disturbances of conductive networks [14]. However, it can be seen that the present sensors showed stable gauge factors even when stretched up to high strain amplitude (i.e., 500%). Based on these piezoresistive sensing test results, it can be concluded that the fabricated sensors, with a wide working range and superior sensitivity, showed high potential for use in applications where both excellent flexibility and sensitivity are required.

### 3.3. Monitoring Movements of Human Fingers

**Figure 7:**
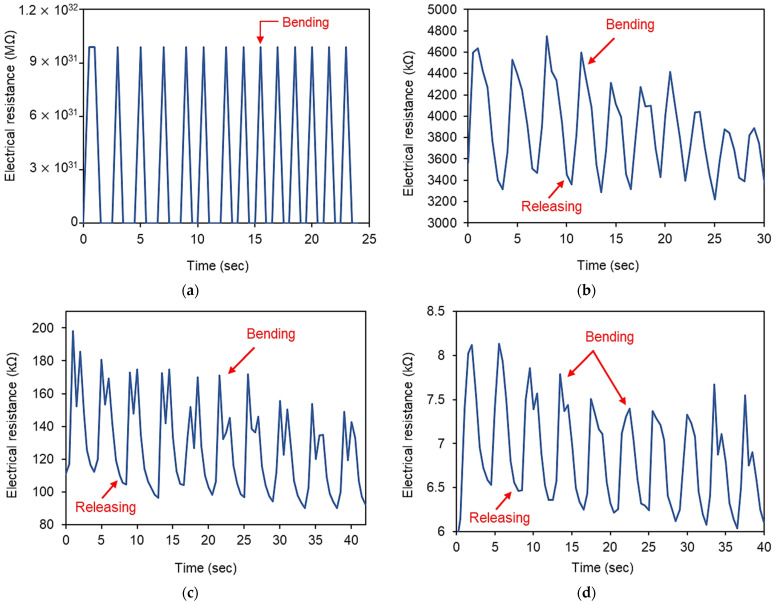
Electrical resistance of composites upon finger bending: (**a**) C1.5; (**b**) C2; (**c**) C3; (**d**) C4.

In this section, the sensors were subjected to the index finger with bending and releasing motions to observe the applicability of using them as human-motion-monitoring sensors, and the results are plotted in Figure 7. During finger bending, C1 sensors showed unstable electrical responses. Its resistance frequently moved outside the measurable range of the multimeter, even at a slight finger bending, which can be attributed to the high resistivity value of this specimen as discussed previously. Moreover, the C1.5 sensor also showed a complete loss of electrical paths upon full bending of the finger, which was recovered upon releasing (Figure 7a). The other sensors (C2, C3, and C4) exhibited measurable changes in electrical resistance in the range of 22–69% upon finger bending, as can be seen in Figure 7b–d.

Upon finger bending, all the sensors showed slight decreases in resistance at peaks. This can be explained by the shoulder phenomenon exhibited by the sensors incorporating carbon-based conductive particles [40]. This type of shoulder phenomenon is reported to be due to the reconstruction, re-distribution, and simultaneous destruction of the electrically conductive network [41]. In addition, the properties of the utilized polymer (i.e., Ecoflex) with super stretchability can also lead to the deformation of their shape as the cyclic loading and/or strain is applied to the composites. Thus, the authors are supposed to add different types and sizes of conductive fillers compared to CNTs to improve the sensing stability of CNT-embedded sensors in future work. However, it can be said that the C2 sensor exhibited a comparably stable sensing response, indicating that a CNT content of 2% is favorable for these sensors with various CNT contents. These test results can also be supported by the piezoresistive sensing mechanisms, which state that the composites with a CNTs amount in the percolation threshold range show higher sensitivity compared to the composites with CNTs contents outside of the percolation threshold range [11]. As a result, the small strain/stress applied to the CNT-embedded composites can significantly alter the CNTs conductive networks, leading to dramatic changes in electrical resistivity [11]. The electrical stability against varying voltages (shown in Figure 4) and resistance changes against varying strain amplitudes (shown in Figure 6) further endorse this fact.

## 4. Conclusions

In this study, flexible polymeric sensors with different CNTs contents (0.5, 1, 1.5, 2, 3, and 4%) were fabricated using the three-roll milling machine method. The percolation threshold of the fabricated sensors was found, and their electrical stability under DC and AC signals was examined. In addition, the piezoresistive sensing performances of the sensors against cyclic tensile loadings were determined, and finger bending/releasing motions were investigated to validate the possibility of using them as human-motion-monitoring sensors. The following conclusions can be drawn based on the experimental results:

1. The percolation threshold of these sensors was approximately 2% as the conductivity drastically increased between 1 and 2% CNTs content. Therefore, the C0.5 specimens with 0.5% CNTs showed no conductivity at all.
2. The electrical stability results indicated that the sensors conform to Ohm’s law. The sensors with a higher amount of CNTs showed excellent electrical stability.
3. The electrical impedance of sensors decreased with the increase in input frequency. However, this variation was also minimized with the increase in CNTs content.
4. The C1 and C1.5 sensors, with 1 and 1.5% CNTs contents, showed a complete loss of electrical paths at the applied strain amplitudes of 50% and 200%, respectively, since their CNTs content was less than the percolation threshold of these sensors.
5. Sensors with 2 to 4% CNTs content showed distinct measurable resistance changes up to 500% strain amplitude, which shows that these sensors can be used for a wide range of high strains.
6. Among all the sensors, the C2 sensor showed the highest sensitivity with gauge factors ranging from approximately 1.79 for 50% strain to approximately 37.04 for 500% strain.
7. Sensors with 2 to 4% CNTs content also exhibited high sensitivity against finger bending/releasing motions, which validates their potential for human-motion-sensing applications or use in wearable devices.

## Figures and Tables

**Table 1:** Details of CNT-embedded composite sensors.

Designation	CNT (wt.%)	Silicone Polymer (g)	CNT (g)
C0.5	0.5	80	0.4
C1	1	80	0.8
C1.5	1.5	80	1.2
C2	2	80	1.6
C3	3	80	2.4
C4	4	80	3.2

**Table 2:** Gauge factor of the sensors under the various applied strain amplitudes.

Specimen	Applied Strain Amplitude (%)					
	50%	100%	200%	300%	400%	500%
C2	1.79	2.58	5.97	11.65	24.20	37.04
C3	0.8	1.06	1.58	1.95	2.16	2.34
C4	0.36	0.73	1.37	1.55	1.67	1.74

## Data Availability

The data presented in this study are available on request from the corresponding author.
